# Primordial odontogenic tumor: a case report and literature review

**DOI:** 10.1186/s13000-019-0867-4

**Published:** 2019-08-17

**Authors:** Qiaochu Sun, Jae-Seo Lee, Okjoon Kim, Young Kim

**Affiliations:** 1 Department of Oral Pathology, School of Dentistry, Chonnam National University, 77 Yongbong-ro, Buk-gu, Gwangju, 61186 Republic of Korea; 2 Department of Oral and Maxillofacial Radiology, School of Dentistry, Chonnam National University, 77 Yongbong-ro, Buk-gu, Gwangju, 61186 Republic of Korea

**Keywords:** Primordial odontogenic tumor, Odontogenic tumors, Odontogenesis

## Abstract

**Background:**

A primordial odontogenic tumor (POT) is a rare, benign, mixed epithelial and mesenchymal odontogenic tumor that has been included as a new entity in the latest World Health Organization (WHO) classification (2017). POT consists of dental papilla-like myxoid connective tissue covered with a delicate membrane of ameloblastic epithelium. Only 15 cases have been documented worldwide, and here, we report the sixteenth case and the first one of South Korea.

**Case presentation:**

An asymptomatic lesion was discovered as an incidental radiographic finding in a 10-year-old boy. The patient had no complaints about the lesion. Cone-beam computerized tomograms revealed a round cavity with a defined cortical border measuring approximately 5 × 5 × 5 mm in size. The lesion was a POT. The patient was treated with enucleation. The tumor showed no recurrence for one year.

**Conclusion:**

This is the first report of POT in South Korea using the novel diagnosis of POT after it was recognized and defined in the latest WHO classification. This novel diagnosis will be useful for pathologists and clinicians in diagnosing and differentiating this new and rare disease from other odontogenic tumors.

## Background

A primordial odontogenic tumor (POT) is a new entity classified as a benign, mixed odontogenic tumor in the fourth edition of the World Health Organization (WHO) classification of Head and Neck Tumors in 2017 [1]. Mosqueda-Taylor et al. (2014) analyzed the clinicopathological and immunohistochemical features in a series of six cases that did not fulfill the previous criteria for odontogenic tumors [2], and the term “primordial odontogenic tumor” was first used to describe the novel lesion.

To date, most cases of POT were found as well-defined unilocular or multilocular radiolucent lesions adjacent to the crown of an unerupted tooth. Patients showed asymptomatic bone swelling, producing root resorption, and buccal or lingual cortical expansion. Macroscopically, the tumor is a pale, slippery, solid nodule that tends to be encapsulated [2, 3]. Histopathologically, POTs consist of variably cellular-to-loose fibrous tissue with dental papilla-like areas, entirely enveloped in a cuboidal-to-columnar epithelium and resembling the inner epithelium of the enamel organ [2]. Bologna-Molina et al. investigated the possible histogenesis and biological behavior of POTs using various immunohistochemical methods and suggested that POT is a benign, odontogenic tumor that develops during the primordial stage of tooth development [4].

Until now, only 15 cases have been documented worldwide [5–7]. To better understand this novel entity to diagnose it correctly, we report the sixteenth case worldwide and the first case of POT in Korea since it was defined in the latest WHO classification.

## Case presentation

A 10-year-old healthy boy visited the Department of Pediatric Dentistry, Chonnam National University in July 2018 to complete root canal therapy. An asymptomatic lesion was discovered incidentally in a conventional panoramic X-ray. There was no history of trauma to the area and he had no complaints about the lesion. There were no abnormal findings in either the physical examination or laboratory data.

**Fig. 1:**
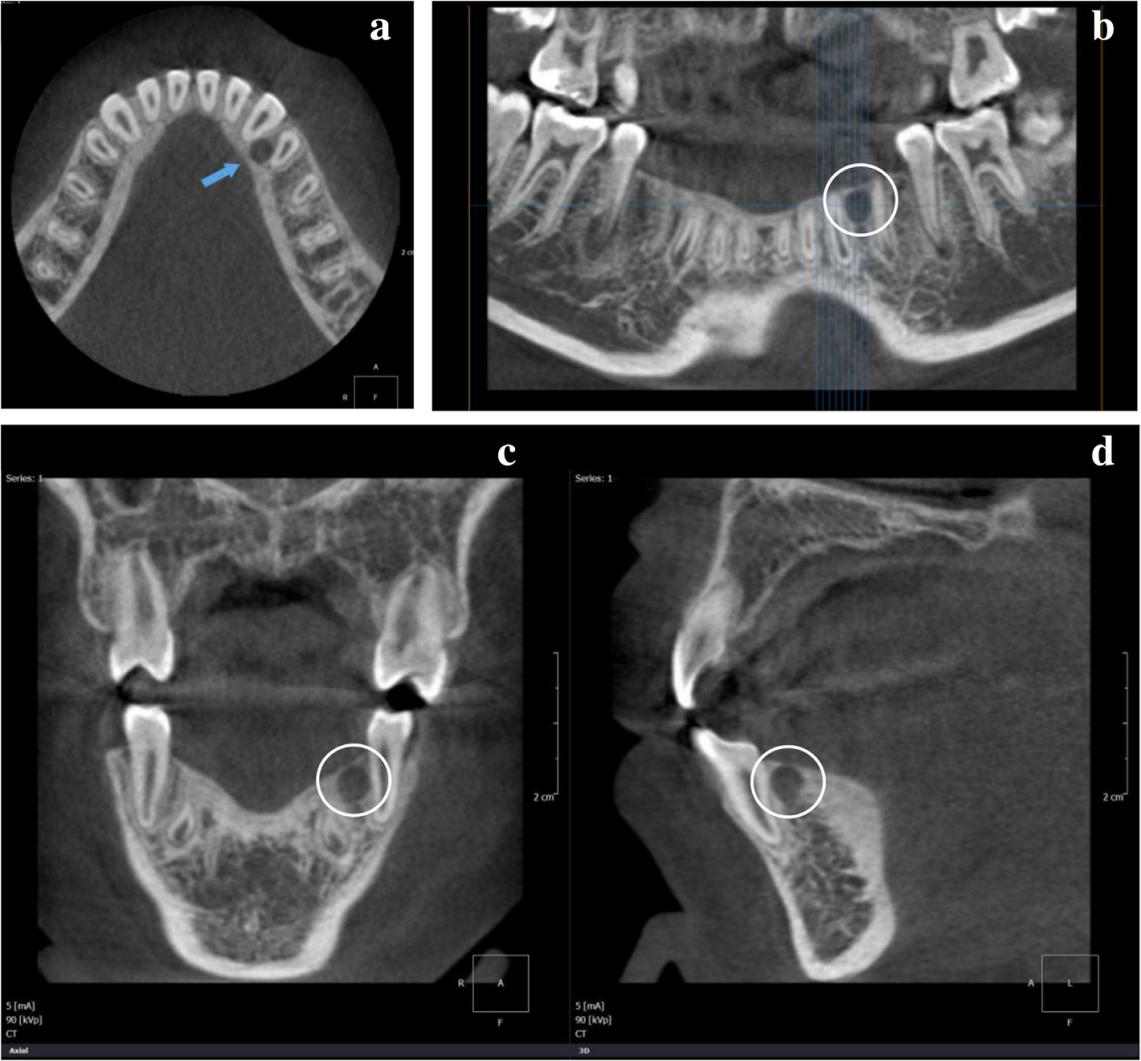
Radiographic findings of POT. **a** Cone-beam CT showing a round cavity with defined cortical border that is mesiolingual to the root of tooth 34. **b-d** Panoramic radiograph demonstrates well-defined radiolucency (circled area) in the mandibular left region

Cone-beam computerized tomograms depicted a round cavity with a defined cortical border measuring approximately 5 × 5 × 5 mm in size, mesiolingual to the root of tooth 34 (Fig. 1a). A panoramic radiograph showed a periapical bone resorption with sclerosing osteitis on the apical to the adjacent tooth (Fig. 1b).

The diagnostic hypotheses from radiology were simple bone cyst, periapical cemental dysplasia, and paradental cyst because of its location and radiologic features. A tumor enucleation was performed, and a whitish, firm, myxoid connective tissue was transferred for pathological analysis. Based on the histopathological study results, POT was confirmed as a definitive diagnosis. There were no adverse events neither signs of recurrence after surgery during a one-year follow-up.

**Fig. 2:**
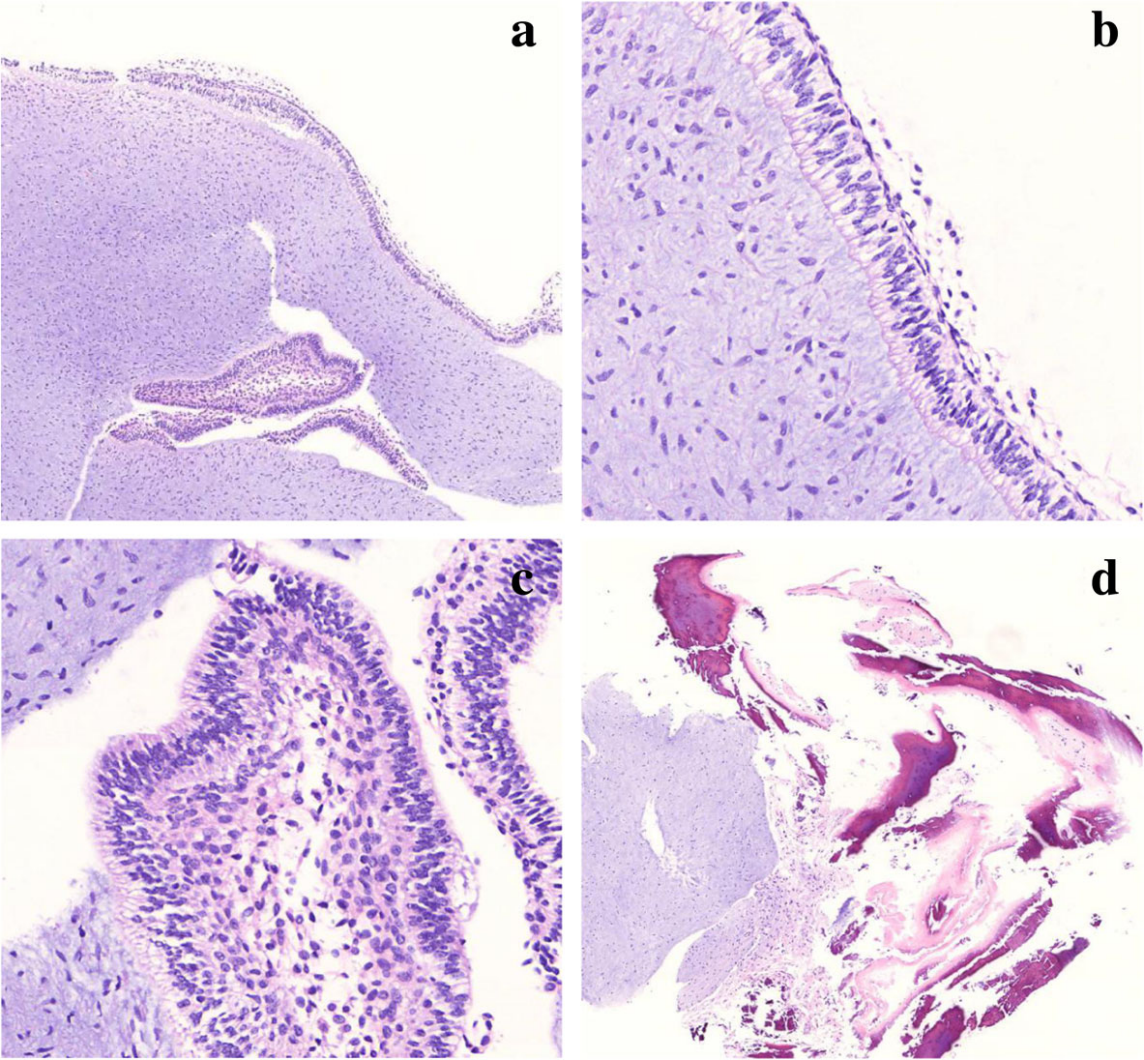
Microscopic findings of the POT. **a** It consisted of a proliferation of cellular myxoid connective tissue, which is less vascular, less cellular, and more collagenous. The periphery of the tumor is surfaced by a monolayer of columnar epithelium. The cord-like or nests of enfolded epithelium are present (hematoxylin and eosin stain; magnification, × 40). **b** The external aspect of the tumor is surfaced by columnar epithelial cells, which show “reverse nuclear polarization” (nuclei displaced away from the connective tissue and cytoplasm showed vacuolated at the bottom part) (magnification, × 200). **c** The cord-like or nests of the enfolded epithelium possessed stellate reticulum between the columnar cells (magnification, × 200). **d** Dentine existed adjacent to the tumor (magnification, × 40)

The gross specimen showed a 5 × 5 × 5-mm-sized well-defined and slippery white nodule of pale, translucent, firm, myxoid connective tissue. Histologically, the periphery of the tumor was enveloped by a delicate membrane of ameloblastic epithelium, which is a single layer of columnar epithelium exhibiting typical “reverse nuclear polarization,” i.e., displacement of nuclei away from the basement membrane and vacuolated cytoplasm at the bottom part. Most of the tumor was composed of loose and myxoid fibrous tissue, including spindle cells (Fig. 2a, b). In some areas, the cords or islands of the epithelium were observed in the connective tissue because of tangential folded sectioning. The cord-like or nests of the enfolded epithelium possessed stellate reticulum between the columnar cells (Fig. 2c). Dentine was found in the peripheral portion of the connective tissue (Fig. 2d), which represents an association between tumor and adjacent tooth.

**Fig. 3:**
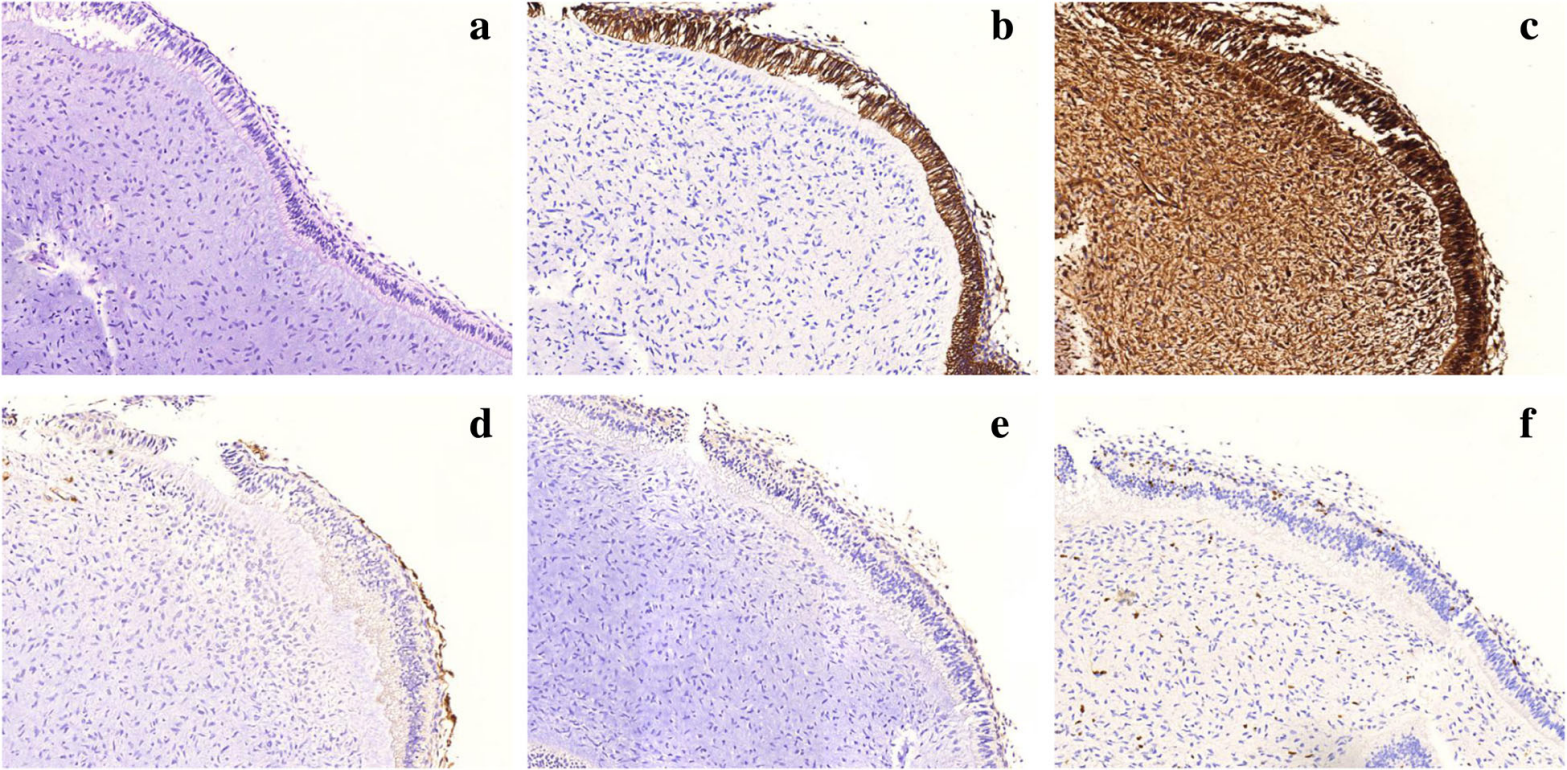
Histopathological and immunohistochemical findings of POT. **a** Hematoxylin and eosin staining of POT. **b** CK19 was positive only in columnar epithelium. **c** Vimentin was positive throughout the tumor tissue (strongly positive in epithelial layers and moderately positive in mesenchymal tumor cells). **d** Ectomesenchymal cells were negative for α-SMA. **e** Ectomesenchymal cells were negative for S-100 protein. **f** Ki67 labeling index was basically lower than 2%. (magnification, × 100)

Upon immunohistochemistry analysis, the epithelial component demonstrated strong positivity for cytokeratin 19 only in columnar cells, which was consistent with previously reported cases. Vimentin was also positive throughout the tumor tissue; specifically, strongly positive in the epithelial layers and moderately positive in mesenchymal tumor cells. In addition, alpha smooth muscle actin (α-SMA) and S100 protein were negative in the ectomesenchymal cells. Ki67 expression was lower than 2% (Fig. 3).

## Discussion and conclusions

POT is a new neoplastic entity, classified as a benign, mixed epithelial and mesenchymal odontogenic tumor in the fourth edition of the head and neck WHO blue book in 2017 [1]. The term “POT” was first established by Mosqueda-Taylor et al. in 2014 [2], and the authors reported a series of six cases clinicopathologically and immunohistochemically.

Until now, there had been only 15 cases of POT reported in the literature. The previously reported cases share similar clinical and radiological findings, which are shown in Table 1. Regarding the statistics of all of these cases, including the current one, there were ten males (62.5%) and six females (37.5%). The median age was 11.3 years for all 16 cases (ranging from 2 to 19 years). The dentition stages of the patients are as follows: five cases (31.25%) affecting the deciduous dentition stage (2–5 years), two cases (12.5%) during mixed dentition stage (8–10 years), and nine cases (56.25%) in the permanent dentition stage (13–19 years). Most cases, including this case (14/16, 87.5%), occurred in the mandible, and the remaining two cases occurred in the maxilla. The prognosis of all POTs was excellent after surgery, except for two cases which were lost to follow-up, recurrences of all reported cases have not been reported to date (median follow-up years = 4.53 ± 6.09, ranging from 3 months to 20 years). In our case, until now after enucleation, there was no recurrence either. It seems that enucleation and extraction of involved tooth were effective treatments because the peripheral columnar epithelium or fibrous pseudocapsule of the tumor clearly delimited the boundaries of the tumor from adjacent tissues. It is worth noting that the geographic regions of the POT cases were mainly located in North and South America (68.8%). Only 25% of the reported cases (including the current case) occurred in Asia, and there was only one case reported in Egypt, Africa. POTs may occur at a higher incidence in Western countries, but greater numbers of cases are needed to further demonstrate this and study the etiology of POTs.

**Table 1:** Summary of previous and current reports of primordial odontogenic tumor

Case No.	Age (years)/gender	Location	Clinical findings	Radiographic findings	Treatments	Follow-up	References
1	18/M	Mandible	Asymptomatic, buccal swelling. Clinically evident for 6 months	RL, UL, well-defined, 45 × 40 mm	Enucleation and tooth extraction	20 years, NED	Mosqueda-Taylor et al. (2014) [2]
2	16/M	Mandible	Asymptomatic, buccal and inferior mandibular cortical bone expansion. Clinically evident for 4 months	RL, UL, well-defined, 55 × 50 mm	Enucleation and tooth extraction	Follow-up lost	Mosqueda-Taylor et al. (2014) [2]
3	16/M	Mandible	Asymptomatic, buccal swelling. Clinically evident for 1 year	RL, UL, well-defined, 65 × 50 mm	Enucleation and tooth extraction	10 years, NED	Mosqueda-Taylor et al. (2014) [2]
4	3/F	Mandible	Asymptomatic, buccal and lingual bony expansion. Clinically evident for 22 months	RL, biloculated, well-defined, 90 × 70 mm	Enucleation and tooth extraction	9 years, NED	Mosqueda-Taylor et al. (2014) [2]
5	13/F	Mandible	Asymptomatic, buccal swelling. Clinically evident for 4 months	RL, biloculated, well-defined, 80 × 50 mm	Enucleation and tooth extraction	3 years, NED	Mosqueda-Taylor et al. (2014) [2]
6	3/F	Maxilla	Asymptomatic, buccal and palatal bony swelling. Clinically evident for 3 months	RL, UL, well-defined, 35 × 30 mm	Enucleation and tooth extraction	6 months, NED	Mosqueda-Taylor et al. (2014) [2]
7	19/M	Mandible	Asymptomatic, buccal and lingual swelling	RL, UL, well-defined, 25 × 19 mm	Excision and tooth extraction	7 months, NED	Slater LJ et al. (2016) [3]
8	8/F	Maxilla	Asymptomatic, buccal swelling	RL, UL, well-defined, 16 × 15 mm	Enucleation	16 months, NED	Ando et al. (2017) [8]
9	5/M	Mandible	Asymptomatic, buccal swelling	RL, UL, well-defined, 80 × 80 mm	Excision and tooth extraction	7 months, NED	Mikami et al. (2017) [9]
10	17/M	Mandible	Asymptomatic, swelling	RL, multilocular, well-defined, 30 × 20 mm	Enucleation and tooth extraction	6 months, NED	Bajpai and Pardhe (2018) [10]
11	15/F	Mandible	Slight fullness of the right mandibular vestibule	RL, multilocular, well-defined, 35 × 20 mm	Excision and tooth extraction	3 months, NED	Asma Almazyad et al. (2018) [11]
12	18/M	Mandible	Asymptomatic, incidentally noted intra-osseous lesion	RL, UL, well-defined, 12 × 7 mm	Curettage and tooth extraction	20 months, NED	Asma Almazyad et al. (2018) [11]
13	2/M	Mandible	Asymptomatic, swelling	RL, multilocular, well-defined, 30 × 40 mm	Excision and tooth extraction	2 years, NED	Hatem Amer et al. (2018) [5]
14	4/M	Mandible	Asymptomatic, buccal and lingual bony expansion. Clinically evident for 8 months	RL, UL, well-defined, 30 × 20 mm	Enucleation and tooth extraction	Follow-up lost	Bomfim B B et al. (2018) [6]
15	13/F	Mandible	Asymptomatic, volume augmentation. Clinically evident for 3 months	RL, UL, well-defined	Enucleation and tooth extraction	13 years, NED	Teixeira L N et al. (2019) [7]
16	10/M	Mandible	Asymptomatic	UL, well-defined, 5 × 5 mm	Enucleation	one year, NED	Present case

*NED* no evidence of disease, *RL* radiolucent, *UL* unilocular

**Fig. 4:**
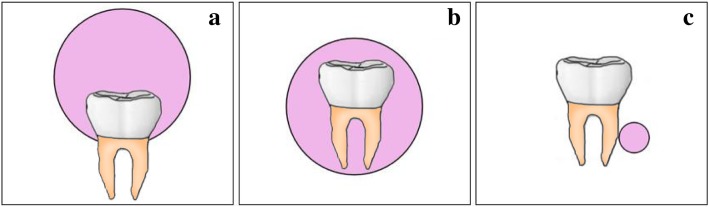
Schematic overview of the POT location and involved tooth. **a** Type A, POT has a pericoronal location in a dentigerous relationship. **b** Type B, the tumor appears to completely envelop an embedded tooth. **c** Type C, the POT is in close proximity to the root of the tooth

The present case showed a rare location with a small tumor, whereas clinical, radiological, and pathologic findings are similar to those of the previous reported cases. Interestingly, the location of this tumor was near the root of the tooth, whereas previously reported cases presented as a pericoronal location in close association with unerupted teeth. To verify a relationship between the patient’s dentition stage and the location of the tumor, we summarized the location of all POTs and listed the information in a schematic diagram. We then attempted to classify the location of POT by three types based on the previous literature (Fig. 4): Type A, the POT has a pericoronal location in a dentigerous relationship; Type B, the tumor appears to completely envelop an embedded tooth; and Type C, the POT is in close proximity to the root of the tooth. There were 12 Type A cases; four of them (33.3%) were in the stage of deciduous dentition, one of them (8.3%) was in the mixed dentition stage, and the remaining seven (58.3%) were in the permanent dentition stage. Three cases fit the criteria for Type B; one of them was in the deciduous dentition stage, and the other two cases were in the permanent dentition stage. Only one case was Type C (the present case), and it was found in the mixed dentition stage (Table 2). In every dentition stage, Type A was the most common. The current case is a unique report of the first Type C case worldwide. It appears that the patient’s dentition stage is not determined by the location type of the POT, although it will be necessary to evaluate more cases. In addition, the POT size in the present case was the smallest compared with that in previously reported cases (ranging from 12 mm to 90 mm) (Table 1). To determine whether there is a relationship between the size of the POT and the location type, we analyzed the size of every case classified by location type as described above. The size of Type A POT ranged from 12 mm to 90 mm and Type B ranged from 25 mm to 80 mm. There is only one case of Type C POT (the present case), and its size was 5 mm. Further evaluation is needed to determine whether a Type C tumor characteristically shows a smaller size than that of Type A and Type B. The number of reported cases is not large enough, therefore, greater numbers of POT cases are required to obtain a better understanding of this rare entity.

**Table 2:** Relationship of different POT types and dentition stage

Type	Deciduous dentition stage	Mixed dentition stage	Permanent dentition stage	Total cases No.
A^a^	4/12 (33.3%)	1/12 (8.3%)	7/12 (58.3%)	12
B^b^	1/3 (33.3%)	0/3 (0%)	2/3 (66.7%)	3
C^c^	0/1 (0%)	1/1 (100%)	0/1 (0%)	1

^a^Type a, POT has a pericoronal location in a dentigerous relationship

^b^ Type B, the tumor appears to completely envelop an embedded tooth

^c^ Type C, the POT is in close proximity to the root of the tooth

In histological findings, our case was enveloped by a single layer of columnar epithelium exhibiting typical “reverse nuclear polarization”, which is known as ameloblastic epithelium. In some areas, the cord-like or islands of the enfolded epithelium possessed stellate reticulum between the columnar cells. Calcification was found in the peripheral portion of the myxoid connective tissue, which represents an association between the tumor and adjacent tooth. The pathological characteristics in our case are conclusive enough to make a diagnosis [3, 8].

According to Mosqueda-Taylor et al. [2], who first described and termed this new entity, POT should be carefully distinguished from an ameloblastic fibroma (AF), central odontogenic fibroma (COF), and odontogenic myxoma (OM). An AF can be easily differentiated from POT because the histological picture is quite different. An AF is a tumor composed of odontogenic ectomesenchyme resembling the dental papilla with epithelial cords and immature mesenchymal stroma without dental hard tissues [12]. POT also contains a small number of cord-like epithelium or nests, which is similar to an AF; however, the epithelial cords or nests of POTs are present in a limited area near the periphery of the tumor [8]. Moreover, the mesenchymal components in AF cases are more cellular, and the ameloblastomatous epithelial component is conspicuous [2, 13, 14], whereas ameloblastomatous islands are not seen in the main body of POTs [11]. A COF is an infrequently reported tumor accounting for only 0.1% of all odontogenic tumors [15–18]. When comparing the pathological features of both entities, COFs have been categorized into two types: an epithelium-poor (simple type) and an epithelium-rich type (WHO type) [18], but it does not show an external covering of ameloblastic epithelium as in POTs. An OM is a rare, benign tumor of odontogenic mesenchymal origin. Radiographically, OMs present as a frequently multilocular radiolucent lesion [19]. The “soap-bubble, honeycomb, or tennis-racket trabeculation” radiological image can be found in OMs [20–25], but the association between OMs and impacted teeth are rarely found to differ from POTs [2–5, 8, 10, 11, 26]. Pathologically, unlike POTs, an OM is never enveloped by ameloblastic epithelium [3].

Among the 16 cases (including this case), only 11 cases have been analyzed immunohistochemically (Table 3). Specifically, 10 cases underwent immunohistochemical analysis for CK19, and in all cases, CK19 was positive in the epithelium of the tumor (predominated in the columnar epithelium). Nine cases were analyzed for vimentin, α-SMA, and S-100 protein, and in 9/9 (100%) of the cases, vimentin was positive throughout the tumor tissue, while α-SMA and S-100 protein were not expressed. Eight cases were analyzed for Ki67, which was lower than 2%. CK19 is often positively expressed in the epithelium of odontogenic cysts and tumors, especially in preameloblasts and secretory ameloblasts [27]. In both the previously presented and current POT cases, CK19 predominated in cubic and columnar epithelial cells [2, 9], suggesting that these epithelial linings express diverse degrees of maturation. This can be supporting evidence that the tumor originated from primordial cellular components of the enamel organ [4]. Vimentin was variably positive in cells of mesenchymal origin [28]. In the present case, vimentin was strongly positive in epithelial cells and moderately positive in mesenchymal cells. Kero et al. [29] demonstrated that vimentin was positively expressed in dental epithelium of the enamel organ between the tenth and twentieth gestational week, but there was no detectable vimentin expression after 27 gestational weeks [30]. These data suggest that the POT may occur at the primordial stage of tooth development around the tenth-twentieth week (cap-to-bell stage). Besides, the presence of the transcription factor PITX2 in POT tumoral epithelium, also supports the hypothesis that this tumor probably derived from the early stages of dental morphogenesis [4]. Teixeira L N et al. [7] mentioned that the expression pattern of cytokeratin 18 in the inner enamel epithelium-like epithelium of POT and that of vimentin in the whole tumor might be important to investigate tumor pathogenesis. This pattern of cytokeratin 18 and vimentin are also observed during tooth development, which reinforces the theory that POT is derived from a primordial tooth germ [7]. Consistent with previously reported cases, α-SMA and the S-100 protein were negative in mesenchymal tumor cells [2, 8, 9]. To quantify the proliferative activity of the tumor, Ki67, which is a marker associated with cell proliferation, was detected. In both our case and in previous studies [2, 4], the expression of Ki67 was low (< 2%) in both epithelial and mesenchymal cells, which is similar to other benign odontogenic tumors, such as OMs [31].

**Table 3:** Immunohistochemistry results of the previous cases and current case

Position	Antibody	Immunohistochemistry results	Positive^a^ (%)
Epithelial cells	CK19	Positive (mainly in columnar epithelium)	10/10 (100%)
	Vimentin	Positive	9/9 (100%)
Mesenchymal tumor cells	Vimentin	Positive	9/9 (100%)
	α-SMA	Negative	0/9 (0%)
	S-100 protein	Negative	0/9 (0%)

^a^.10 cases were analyzed for CK19 using immunohistochemical analysis, and all cases showed positive results in the tumor epithelium. Vimentin was also 100% positive throughout the tumor in all nine cases in which it was analyzed. α-SMA and S-100 protein were negative in all nine cases

We herein report the first case of POT in South Korea since it was newly categorized in the latest WHO classification in 2017. As there are only 16 documented cases, including this report, in English literature worldwide, more clinical, pathological, and radiographical information is needed to further understand the disease entity. The size of the POT reported in our article is smaller than previously reported cases. In most of other previously reported POT cases, the tumor showed an apparent pericoronal position with unerupted tooth [2, 3, 8, 11]. Interestingly, unlike these reports, the location of the POT in this case was adjacent to root of the tooth. Given the rarity of this tumor and the limited information known to date, it is important to report new cases to enlarge the understanding of this condition. We hope that the case proposed here will be useful to diagnose and differentiate this new and rare entity from other odontogenic tumors, as well as to help determine a disease entity.

## Data Availability

All data generated or analysed during this study are included in this published article.

## Abbreviations

AF: Ameloblastic fibroma
COF: Central odontogenic fibroma
OM: Odontogenic myxoma
POT: Primordial odontogenic tumor
WHO: World Health Organization
α-SMA: Alpha smooth muscle actin
